# Case Report: A novel CXCR4 variant (p.S341Y) in a family with a pathogenic NFKB1 variant and variable clinical manifestations

**DOI:** 10.3389/fimmu.2025.1641122

**Published:** 2025-08-20

**Authors:** Melis Yilmaz, Katarina Zmajkovicova, Rahim Z. Miller, Grace Blair, Maryssa Ellison, Boglarka Ujhazi, Maria Chitty Lopez, Joseph F. Dasso, Jacob R. Bledsoe, Krisztian Csomos, Barbara Maierhofer, Adriana Badarau, Joao P. Pereira, Henry Kanarek, Christoph B. Geier, Jolan E. Walter

**Affiliations:** ^1^ Division of Allergy and Immunology, Department of Pediatrics and Medicine, Morsani College of Medicine, University of South Florida at Johns Hopkins All Children’s Hospital, St. Petersburg, FL, United States; ^2^ Research Department, Formerly X4 Pharmaceuticals (Austria) GmbH, Vienna, Austria; ^3^ Division of Allergy and Immunology, Department of Pediatrics, Johns Hopkins All Children’s Hospital, St. Petersburg, FL, United States; ^4^ Department of Pathology, Boston Children’s Hospital, Harvard Medical School, Boston, MA, United States; ^5^ Department of Immunobiology, Yale School of Medicine, Yale University, New Haven, CT, United States; ^6^ Kanarek Allergy, Asthma & Immunology, Overland Park, KS, United States; ^7^ Division of Immunology, Faculty of Medicine and Health Sciences, University Medicine Oldenburg, Oldenburg, Germany; ^8^ Institute of Medical Genetics, Faculty of Medicine and Health Sciences, University Medicine Oldenburg, Oldenburg, Germany

**Keywords:** CXCR4, WHIM, NFKB1, neutropenia, CVID

## Abstract

WHIM syndrome is typically caused by C-terminal gain-of-function variants in *CXCR4*, yet clinical heterogeneity suggests additional genetic modifiers. We investigated a family in which the 22-year-old proband harbored two heterozygous variants: a novel *CXCR4* missense variant, c.1022C>A (p.S341Y), and a frameshift variant in *NFKB1*, c.980dup (p.A328Sfs*12). Functionally, CXCR4 p.S341Y substitution - located two residues upstream of the known pathogenic p.E343K variant - increased CXCL12-induced chemotaxis and ERK/AKT signaling while minimally affecting receptor internalization, supporting a partial *CXCR4* gain-of-function. The *CXCR4* variant co-segregated with mild neutropenia, recurrent respiratory infections, and cutaneous warts in the paternal lineage. In contrast, the maternal *NFKB1* variant was associated with agammaglobulinemia and autoimmunity. Their co-inheritance in the proband resulted in a blended WHIM/CVID phenotype characterized by myelokathexis, B-cell maturation arrest and T-cell dysregulation. This case expands the phenotypic spectrum of *CXCR4* variants and highlights how multilocus inheritance can obscure classical diagnostic boundaries and guide individualized therapy.

## Introduction

Warts–hypogammaglobulinemia–infections–myelokathexis (WHIM) syndrome is a rare inborn error of immunity (IEI) characterized by chronic neutropenia, recurrent bacterial and human papillomavirus (HPV) infections, and antibody deficiency (1). In most cases, the underlying genetic defect is a heterozygous gain-of-function (GOF), autosomal dominant variant in C-X-C chemokine receptor 4 gene *(CXCR4)*—typically a C-terminal truncation that impairs receptor internalization leading to amplified signaling in the presence of its endogenous ligand, CXCL12 (2). Clinical phenotype, however, is highly variable. Symptom onset can range from infancy to late adulthood, cytopenias fluctuate over time, and complications such as end organ damage (e.g. bronchiectasis and hearing loss) autoimmunity, or malignancy can arise unpredictably (3).

Genotype–phenotype studies show that the depth of neutropenia, lymphopenia, susceptibility to infection, and extent of myelokathexis generally track with the degree of impaired internalization and downstream excessive signaling. In this context, truncating nonsense or frameshift *CXCR4* variants generally confer more severe disease than missense changes (4, 5). Nevertheless, even individuals harboring the same familial *CXCR4* allele can differ markedly in clinical presentation of disease severity, suggesting additional genetic modifiers (6).

Recent large-scale sequencing studies have shown that 10–20% of patients with inborn errors of immunity carry a second, independent pathogenic variant that modifies their clinical presentation (7). A notable example is *NFKB1* haploinsufficiency, now recognized as the most frequent monogenic cause of common variable immunodeficiency (CVID) (8). Its penetrance is incomplete (~70%), and its clinical spectrum ranges from subclinical, hypogammaglobulinaemia to progressive antibody failure accompanied by autoimmunity, enteropathy, cytopenia, or lymphoid malignancy (9).

Here, we report a kindred whose index case carries two heterozygous variants: a novel *CXCR4* missense variant (c.1022C>A; p.S341Y) adjacent to the known pathogenic p.E343K missense variant (10), and a truncating *NFKB1* frameshift (c.980dup; p.A328Sfs*12). Through integrated immunophenotyping, in-silico modelling and cell-based assays, we demonstrate that p.S341Y confers a partial GOF. In combination with *NFKB1* haploinsufficiency this results in a blended WHIM/CVID phenotype. This pedigree broadens the *CXCR4* variant spectrum, underscores the need for functional validation of variants of uncertain significance (VUS), and illustrates how multi-locus pathogenic variations can blur classical diagnostic criteria.

## Patient case

**Figure 1:**
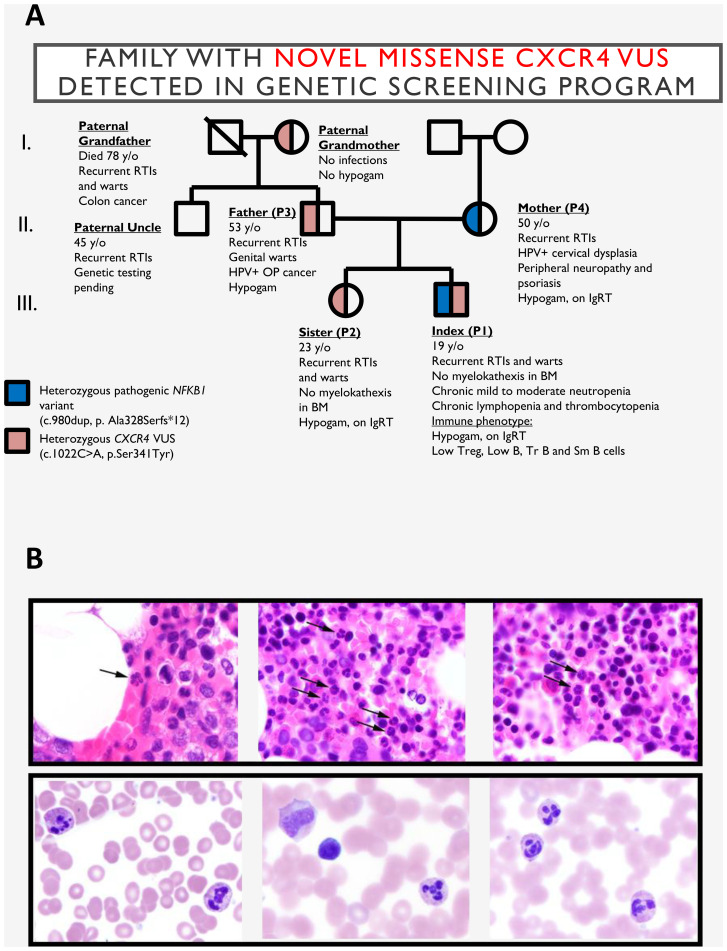
**(A)** Pedigree of the family harboring the CXCR4 p.S341Y variant. **(B)** Top: Bone marrow biopsy from the index patient (P1) demonstrating increased mature segmented neutrophils, including forms with hyperlobated nuclei consistent with myelokathexis (indicated by arrows). Bottom: Bone marrow aspirate smears from the index patient’s sister (P2) showing neutrophils with hyperchromatic, hypermature, and apoptotic chromatin and subtle abnormal nuclear lobation, also consistent with myelokathexis. BM, bone marrow; CXCR4, C-X-C chemokine receptor 4 gene; P1, index patient; P2, index patient’s sister. RTIs, respiratory tract infections; Hypogam, hypogammaglobulinemia; OP, oropharyngeal; TR B, transitional B cells; SM B, class-switched memory B cells; SmB, class-switched memory B cells.

The index patient (P1) is a 22-year-old male under long-term care by hematology and immunology specialist for history of chronic neutropenia, lymphopenia (**Supplementary Figure S1**) and recurrent infections since childhood. These included aphthous ulcers, periodontal disease, oral herpes, otitis media associated with hearing loss, and pneumonia that did not require hospitalization (**Figure 1A**). Additional hematologic findings include thrombocytopenia with frequent epistaxis. At age 17, P1 was diagnosed with CVID and initiated on immunoglobulin replacement therapy (IgRT).

Beginning in elementary school, P1 developed extensive cutaneous warts on the hands and feet, which persisted through adolescence. A reduction in wart burden was observed following the initiation of IgRT in 2017.

Initially, only the mother (P4; 52y, female) had been evaluated for immune dysfunction. She had a history of recurrent infections, neuropathy, myalgia, arthritis, and psoriasis, and was ultimately diagnosed with CVID characterized by agammaglobulinemia. The sister (P2; 26y, female) reported a milder phenotype, consisting of recurrent upper respiratory tract infections and episodic cutaneous warts. She recalled the appearance of 3–4 warts beginning around age 8, which resolved spontaneously or with over-the-counter treatment by age 14.

Considering the familial clustering of immune-related phenotypes, both siblings underwent extended immunologic evaluation, which confirmed antibody deficiency and chronic neutropenia. As a result, both were started on IgRT. While the antibody deficiency accounted for their susceptibility to infection, the cause of persistent neutropenia remained elusive, prompting referral for genetic evaluation.

Genetic testing was conducted through the Path4ward Program (Invitae, San Francisco, CA), sponsored by X4 Pharmaceuticals, using a focused 23-gene panel with the purpose of identifying novel pathogenic variants in patients with history of chronic neutropenia (11). Both P1 and P2 were found to carry a heterozygous *CXCR4* missense variant (c.1022C>A, p.S341Y) classified as a variant of uncertain significance (VUS). Surprisingly, their mother, who was hypothesized to share the same genotype, did not carry this variant. Instead, segregation analysis revealed that the variant was inherited from the paternal lineage: the father (P3; 54 years old)—who had a history of recurrent respiratory infections, genital warts, and HPV-positive oropharyngeal carcinoma—was heterozygous for c.1022C>A, p.S341Y. The variant was also detected in the paternal grandmother, who only reported recurrent sinusitis, suggesting incomplete penetrance.

Given the absence of the *CXCR4* variant in the maternal genome, whole exome sequencing was performed in P1 and identified a heterozygous frameshift variant in *NFKB1* (c.980dup; p.A328Sfs*12), previously reported as pathogenic and associated with *NFKB1* haploinsufficiency. Therefore, P1 was heterozygous for both the novel *CXCR4* variant and the established pathogenic *NFKB1* variant, consistent with a blended immunodeficiency phenotype.

To assess the potential pathogenicity of the CXCR4 p.S341Y variant, bone marrow evaluation was performed in P1 and P2. This revealed subtle but suggestive features of myelokathexis with evidence of neutrophil hypermaturation in P2 (**Figure 1B**). Immunophenotyping further supported complex immune dysregulation: P1, P3, and P4 exhibited reduced frequencies of CD3+, CD4+, regulatory T cells and class-switched memory B cells. All individuals (P1-P4) showed low CD8+ T cells and CD8+ terminally differentiated effector memory T cells (TEMRA) subsets, while P1 and P4 also had marked reductions in transitional and mature naïve B cells. Expansion of CD21^lo^ B and T follicular helper (Tfh) cells in P1, P3, and P4 indicated ongoing immune activation. These findings reflect overlapping features of both *NFKB1* haploinsufficiency and WHIM syndrome, supporting a model of multilocus-driven immunodeficiency (**Supplementary Table S1**).

### *In silico* characterization of the CXCR4 p.S341Y variant

The p.S341Y missense variant is located in the phylogenetically conserved C-terminal region of CXCR4 near the known WHIM-associated variant p.E343K - just two amino acids downstream (**Supplementary Figures S2A-C**). This variant (p.S341Y) has not previously been reported in individuals with *CXCR4*-related disorders and no ClinVar entry existed prior to its identification in this family. It is present at very low frequency in population databases, including GnomAD, TopMED and the NHLBI Exome Sequencing Project (rs148454403, **Supplementary Figure S2D**). *In silico* prediction tools classify the p.S341Y variant as deleterious (SIFT score 0) and probably damaging (PolyPhen score = 0.994).

### *In vitro* characterization of the CXCR4 p.S341Y variant

Given its proximity to known GOF variants and in silico predictions suggesting pathogenic potential, we hypothesized that the CXCR4 p.S341Y variant may alter receptor function. To evaluate this, we transfected CXCR4-negative K562 cells to express either wild-type (WT) CXCR4, the p.S341Y variant, or known WHIM-associated variants p.E343K and p.R334X. These recombinant cells were then assessed using a panel of *in vitro* assays examining CXCR4-mediated responses, including chemotaxis toward CXCL12, downstream ERK and AKT phosphorylation, and receptor internalization kinetics following CXCL12 stimulation.

**Figure 2:**
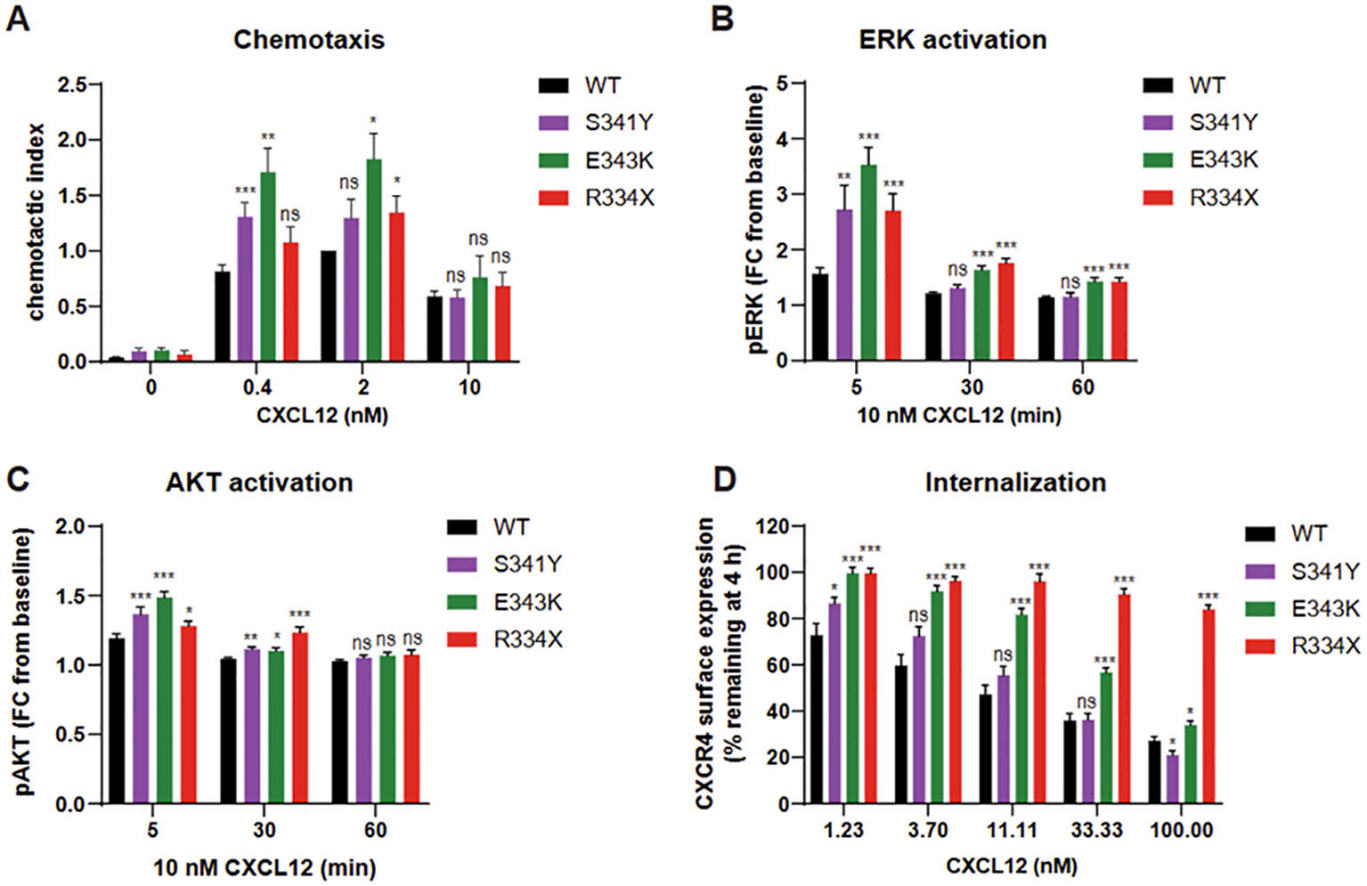
K562 cells expressing CXCR4 wild-type (WT), CXCR4 p.S341Y, p.R334X and p.E343K variants were stimulated with CXCL12 and assessed for chemotaxis **(A)**, ERK and AKT pathway activation **(B, C)** and receptor internalization **(D)**. AKT, protein kinase **(B)**; *CXCR4*, C-X-C chemokine receptor 4 gene; ERK, extracellular signal-regulated kinase; WT, wild type; pAKT, phosphoAKT; pERK, phosphoERK; FC, fold change. *: p<0.05; **: p<0.01; ***:p<0.001; ns: not significant.

Cells expressing p.S341Y exhibited enhanced chemotactic response to CXCL12, though this increase was observed specifically at a concentration of 0.4 nM (**Figure 2A**). This modest chemotactic gain-of-function was accompanied by elevated phosphorylation of ERK and AKT, reaching levels comparable to those induced by the established WHIM variants p.E343K and p.R334X (**Figures 2B, C**). Notably, while p.S341Y elevated the amplitude of ERK activation, the duration of the signal remained transient, unlike the prolonged signaling seen in p.E343K and p.R334X.

Receptor internalization assays revealed distinct functional profiles. The p.E343K and p.R334X variants markedly impaired internalization across a range of CXCL12 concentrations. In contrast, p.S341Y-expressing cells displayed normal internalization at most concentrations, with a significant increase in internalization at low CXCL12 (1.23 nM) and a decrease only at high concentration (100 nM) (**Figure 2D** and **Supplementary Figure S2E**). These findings suggest that the p.S341Y variant confers a partial and context-dependent gain-of-function phenotype, distinct from more severe WHIM-associated variants.

## Discussion

Here, we describe a multidisciplinary approach to determine the pathogenicity of *CXCR4* c.1022C>A, p.S341Y variant, identified in a family with four carriers, including an index case who also harbored a second, pathogenic *NFKB1* variant. Based on the clinical presentation, the index patient met the diagnostic criteria for WHIM Syndrome under the European Society for Immunodeficiencies (ESID) guidelines fulfilling major criteria (chronic neutropenia, myelokathexis) and several supportive criteria (recurrent warts, hypogammaglobulinemia, chronic lymphopenia, and a first-degree relative with neutropenia) (12). In-depth immune evaluation revealed antibody deficiency syndrome with dysregulated B cell development in family members carrying the *CXCR4* variant, whereas P4 – who carried only the *NFKB1* variant – showed a profound antibody deficiency and immunologic markers consistent with CVID. While *in silico* analyses suggested the p.S341Y variant was likely deleterious, they were insufficient to establish pathogenicity, prompting the need for *in vitro* functional studies.

These functional assays revealed a complex picture. Cells expressing p.S341Y exhibited CXCR4 GOF features, including enhanced chemotaxis and increased ERK/AKT activation. However, receptor internalization remained largely intact. This contrasts with classical WHIM-associated CXCR4 variants such as p.R334X, which typically show a triad of impaired receptor internalization, enhanced chemotaxis, and downstream signaling (13, 14). Notably, other variants show divergent patterns: for example, p.E343K variant exhibits mild internalization defects but clear GOF in migration and signaling (10), while p.L317fsX3 impairs receptor internalization but reduced downstream signaling and chemotaxis (15). The landscape of VUS recently includes (p.D84H) of the second transmembrane domain of CXCR4 (16). These qualitative differences may partly explain the broad phenotypic heterogeneity observed in WHIM syndrome.

Clinically, such distinctions appear to correlate with disease severity. Patients with p.E343K often present with milder neutropenia, normal absolute B-cell counts and low T-cells, while patients with p.L317fsX3 may have severe neutropenia, low B-cells and normal T-cell count (10, 15). In this context, the mild functional perturbations associated with p.S341Y suggest a modifier role, potentially resulting in a less severe clinical phenotype than typical WHIM cases. This is supported by the observation that the index patient – who carried both p.S341Y and the pathogenic *NFKB1* variant – had the most pronounced clinical presentation among family members with the *CXCR4* variant alone, raising the possibility of potentiation or additive effects between the two variants. The distinctive genetic context in this family underscores the importance of comprehensive genetic testing for all relatives affected by immunodeficiency. Dual genetic diagnosis within the same patient such as this may occur in 10% to 20% of cases of inborn errors of immunity (IEI). Our findings underscore that even subtle functional changes in immune-related genes can significantly influence clinical expression, particularly in the context of multilocus pathogenic variation.

## Conclusion

In conclusion, characterization of the CXCR4 p.S341Y variant as a disease-modifying susceptibility allele demonstrates how modest functional alterations in receptor signaling can contribute to complex immunophenotypes, particularly when co-inherited with other pathogenic variants, such as *NFKB1* haploinsufficiency. Although p.S341Y lies near known pathogenic CXCR4 variants, its classification had remained uncertain; our findings provide evidence of partial gain-of-function behavior, supporting its role in WHIM-like manifestations. Importantly, the presence of this variant in unaffected individuals, along with its low but detectable population frequency, highlights the critical need to interpret genetic findings within the context of familial segregation, functional data, and multilocus inheritance models. The variable clinical severity observed across family members carrying the same variant underscores the importance of integrated genomic and immunologic evaluation. This case illustrates how overlapping molecular contributions can obscure traditional diagnostic boundaries and reinforces the necessity of personalized, mechanism-driven approaches to diagnosing and managing primary immunodeficiencies.

## Data Availability

The datasets presented in this study can be found in online repositories. The names of the repository/repositories and accession number(s) can be found in the article/Supplementary Material.

## Supplementary material

Supplementary Table 1 Extensive immune phenotyping of P1, P2, P3 and P4 including examination of complete blood count (CBC) with differential, humoral immune parameters (immunoglobulin levels and vaccine titers), cellular immunity (B- and T-cell subsets). CBC, complete blood count; P1, index patient; P2, index patient’s sister; P3, index patient’s father, P4, index patient’s mother; WBCs, white blood cells; IgRT, Immunoglobulin replacement therapy; TEMRA, terminally differentiated effector memory T cells; DN T cells, double negative T cells; TCRab+, T cell receptor alpha/beta positive; Tfh, T follicular helper; SM, switched memory; NK, natural killer.

Supplementary Figure 1
**(A)** Absolute neutrophil count (ANC) measurements for P1 (proband) over a period spanning from 2016 to 2022, demonstrating chronic neutropenia with persistently low ANC values. **(B)** ANC measurements for P2 over a similar timeframe, indicating moderate neutropenia. **(C)** Absolute lymphocyte count (ALC) for P1, highlighting persistent lymphopenia across multiple timepoints. **(D)** ALC for P2, revealing mildly reduced lymphocyte counts compared to reference values. The proband (P1), a 22-year-old male under long-term hematology and immunology care, carries a novel heterozygous CXCR4 variant (c.1022C>A; p.S341Y) associated with enhanced CXCL12-induced signaling and a frameshift NFKB1 variant (c.980dup; p.A328Sfs*12). Together, these variants underlie a blended WHIM/CVID phenotype characterized by myelokathexis, B-cell maturation arrest, and T-cell dysregulation.

Supplementary Figure 2
**(A)** Alignment of CXCR4 C-terminal amino acid sequences (residues 301-352) across indicated species. **(B)** Schematic showing the location of the p.S341Y variant within the CXCR4 protein. **(C)** C-terminal amino acid sequences of CXCR4 WT, p.S341Y, p.R334X and p.E343K, including the known pathogenic variant p.E343K. **(D)** Allele frequency of the p.S341Y variant in publicly available databases. **(E)** Internalization assay of CXCR4 variants: K562 cells were stimulated with CXCL12 and assessed for receptor internalization after 45 minutes. CXCR4, C-X-C chemokine receptor 4; WHIM, warts, hypogammaglobulinemia, infections, and myelokathexis; WT, wild type.
