# Supplementary material for: Case Report: A novel CXCR4 variant (p.S341Y) in a family with a pathogenic NFKB1 variant and variable clinical manifestations
Source: Front Immunol. 2025 Aug 20;16:1641122. doi: 10.3389/fimmu.2025.1641122 (PMC12405443; doi:10.3389/fimmu.2025.1641122)

A

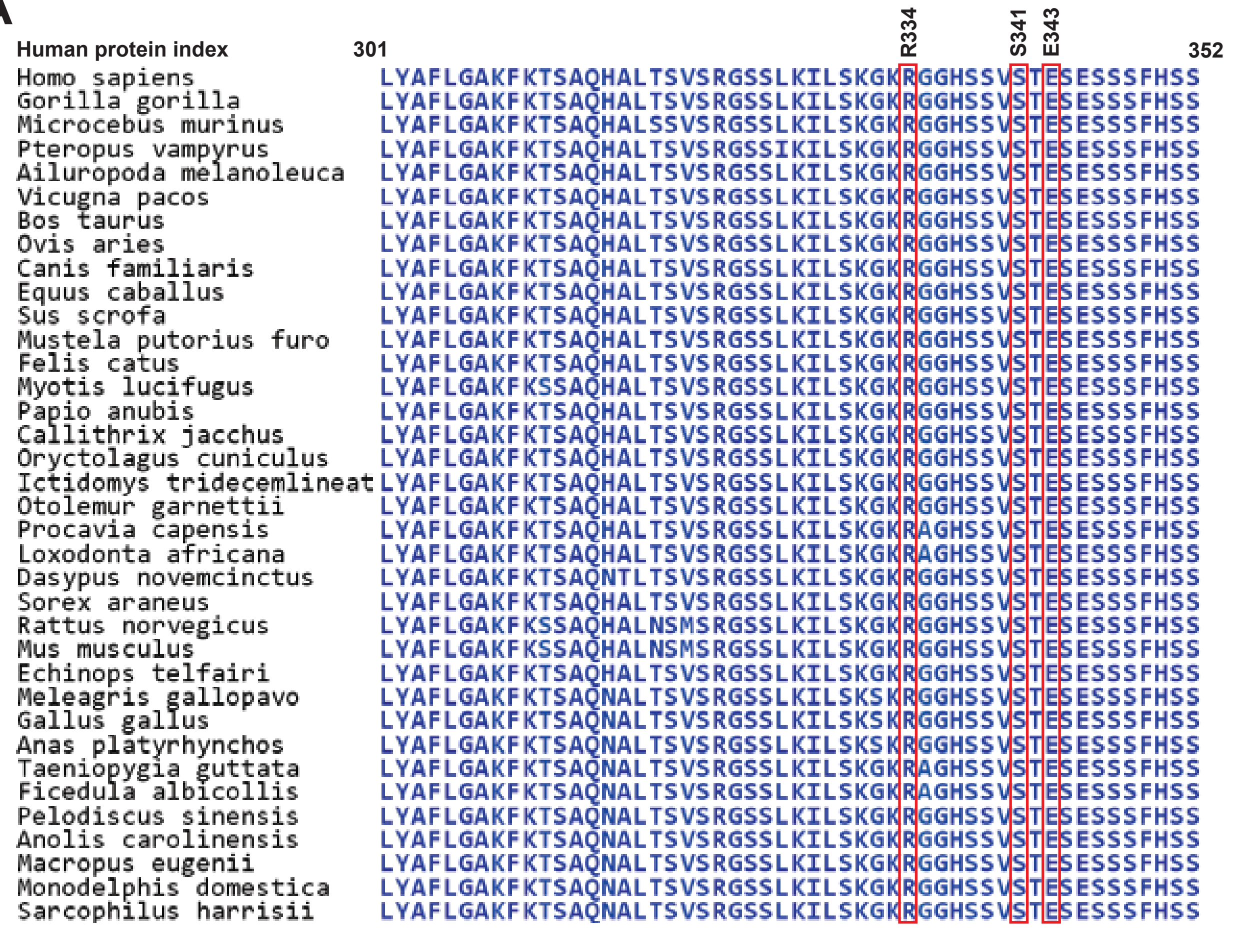

B

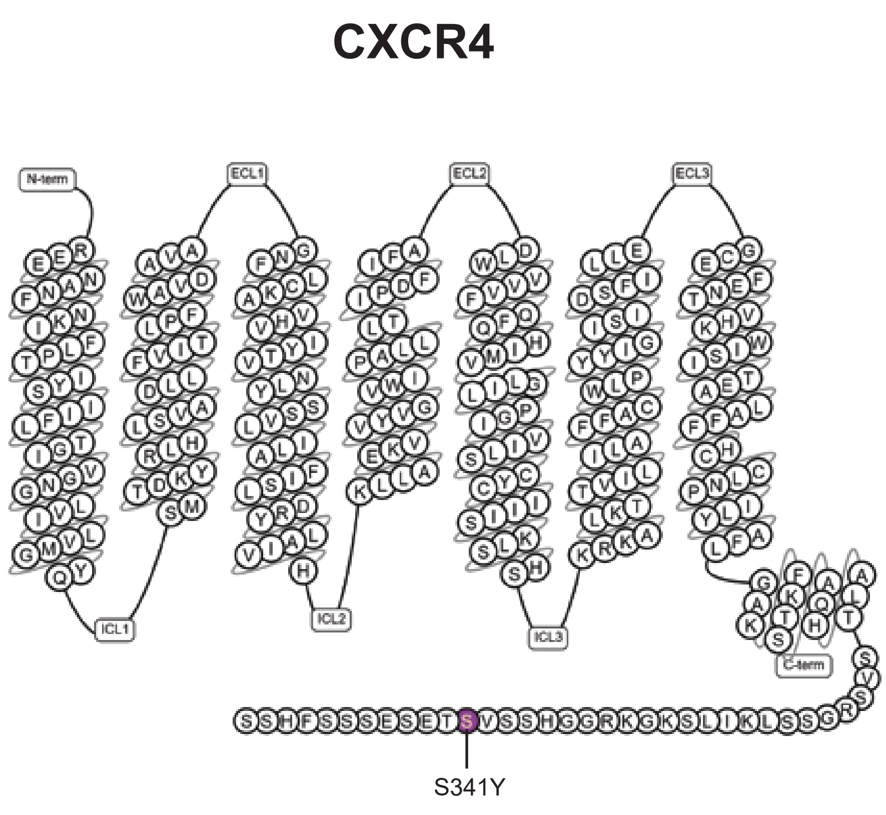

C

WT CXCR4 301 LYAFLGAKFKTSAQHALTSVSRGSSLKILSKGKRGGHSSVSTESESSSFHSS 352

degradation motif

internalization / β-arrestin binding

S341Y	LYAFLGAKFKTSAQHALTSVSRGSSLKILSKGKRGGHSSVYTESESSSFHSS
R334X	LYAFLGAKFKTSAQHALTSVSRGSSLKILSKGK
E343K	LYAFLGAKFKTSAQHALTSVSRGSSLKILSKGKRGGHSSVSTKSESSSFHSS

D

rs148454403 p.S341Y

dataset	AF	no of individuals	heterozygotes	homozygotes
GnomAD_exomes	0.000004	1	1	0
GnomAD	0.000007	1	1	0
ExAc	0.000012	1	1	0
TOPMED	0.000008	2	2	0
GoESP	0.000077	1	1	0

E

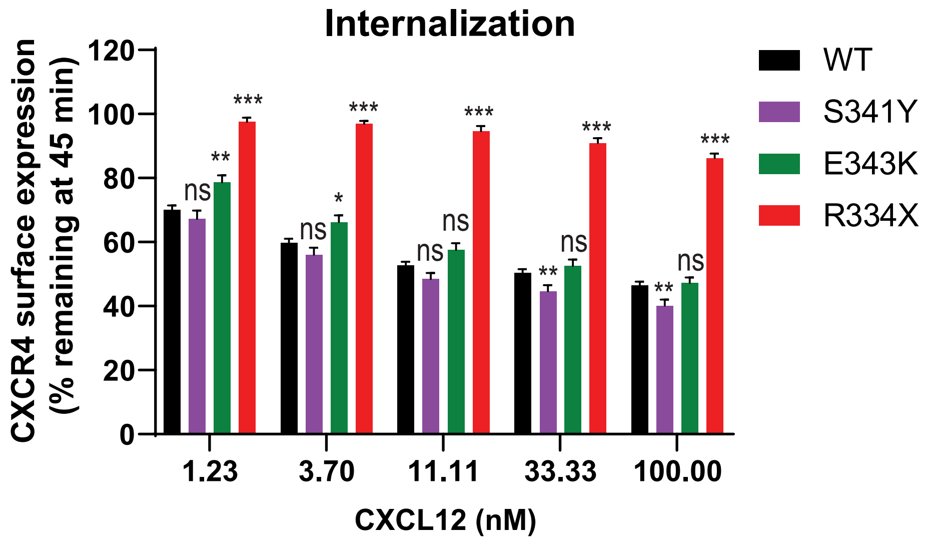

Supplement: Supplementary Figure 1 — (A) Absolute neutrophil count (ANC) measurements for P1 (proband) over a period spanning from 2016 to 2022, demonstrating chronic neutropenia with persistently low ANC values. (B) ANC measurements for P2 over a similar timeframe, indicating moderate neutropenia. (C) Absolute lymphocyte count (ALC) for P1, highlighting persistent lymphopenia across multiple timepoints. (D) ALC for P2, revealing mildly reduced lymphocyte counts compared to reference values. The proband (P1), a 22-year-old male under long-term hematology and immunology care, carries a novel heterozygous CXCR4 variant (c.1022C>A; p.S341Y) associated with enhanced CXCL12-induced signaling and a frameshift NFKB1 variant (c.980dup; p.A328Sfs*12). Together, these variants underlie a blended WHIM/CVID phenotype characterized by myelokathexis, B-cell maturation arrest, and T-cell dysregulation. [file DataSheet1.pdf]
