# Supplementary figures and images for: Case Report: A novel CXCR4 variant (p.S341Y) in a family with a pathogenic NFKB1 variant and variable clinical manifestations
Source: Front Immunol. 2025 Aug 20;16:1641122. doi: 10.3389/fimmu.2025.1641122 (PMC12405443; doi:10.3389/fimmu.2025.1641122)

P1 Proband - ANC Level

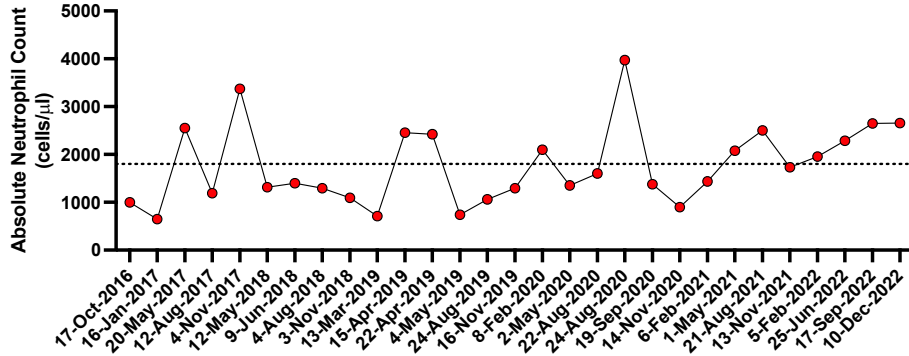

P1 Proband - ALC Level

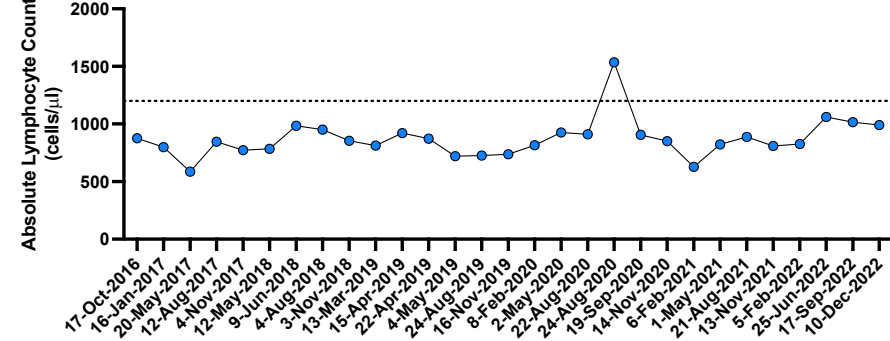

P2 - ANC Level

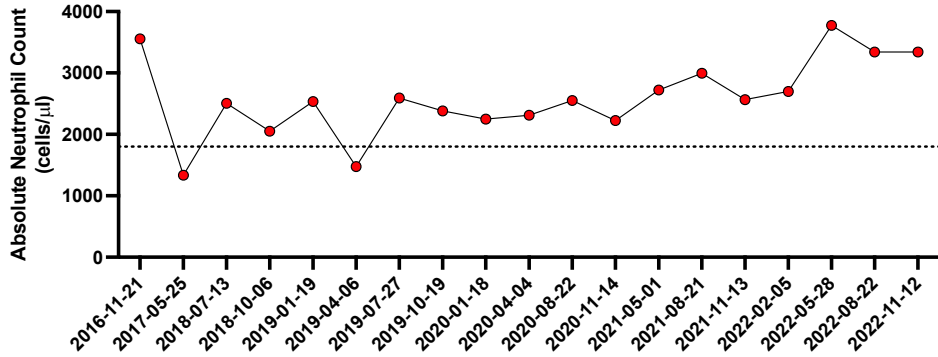

P2 - ALC Level

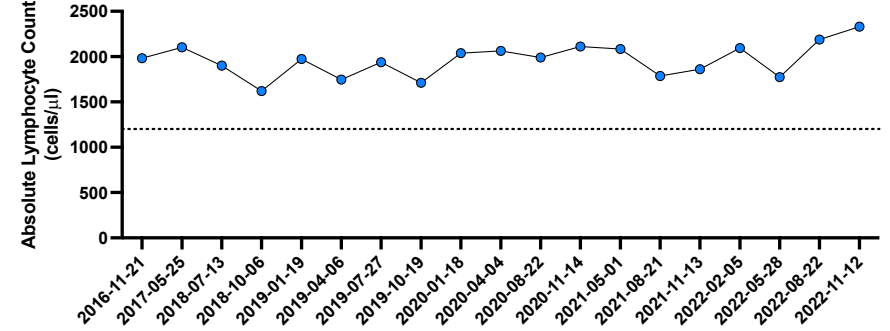

Supplement: Supplementary Figure 2 — (A) Alignment of CXCR4 C-terminal amino acid sequences (residues 301-352) across indicated species. (B) Schematic showing the location of the p.S341Y variant within the CXCR4 protein. (C) C-terminal amino acid sequences of CXCR4 WT, p.S341Y, p.R334X and p.E343K, including the known pathogenic variant p.E343K. (D) Allele frequency of the p.S341Y variant in publicly available databases. (E) Internalization assay of CXCR4 variants: K562 cells were stimulated with CXCL12 and assessed for receptor internalization after 45 minutes. CXCR4, C-X-C chemokine receptor 4; WHIM, warts, hypogammaglobulinemia, infections, and myelokathexis; WT, wild type. [file DataSheet2.pdf]
